# Citizens’ perceptions of the Chilean social outburst: environmental collective action willingness and the role of political identification, democracy, and gender

**DOI:** 10.3389/fpsyg.2026.1736326

**Published:** 2026-03-30

**Authors:** Fuad Hatibovic, Ximena Faúndez, Ketty Cazorla, María Angélica Cruz, Graciela Rubio, Juan Sandoval, José Manuel Gaete, Francisco Sotomayor, Raúl Hozven, María Paz Godoy, Claudia Montero, Omar Sagredo

**Affiliations:** 1 Faculty of Social Sciences, School of Psychology, Universidad de Valparaíso, Valparaíso, Chile; 2 Center for Interdisciplinary Studies on Political Culture, Memory, and Human Rights, University of Valparaíso, Valparaíso, Chile; 3 Faculty of Social Sciences, School of Social Work, Universidad de Valparaíso, Valparaíso, Chile; 4 Faculty of Social Sciences, School of Sociology, Universidad de Valparaíso, Valparaíso, Chile; 5 Faculty of Humanities and Education, Institute of History and Social Sciences, Universidad de Valparaíso, Valparaíso, Chile; 6 Information and Management Control Engineering, Faculty of Economic and Administrative Sciences, Universidad de Valparaíso, Valparaíso, Chile

**Keywords:** democracy, environmental collective action, gender, political identification, social outburst

## Abstract

This study examines citizens’ perceptions of the 2019 Chilean social outburst using cross-sectional survey data from a stratified probabilistic sample of 809 adults in the Valparaíso Region, Chile. We analyse how environmental collective action willingness, political identification, attitudes toward democracy, and gender are associated with evaluations of the protest movement. Descriptive analyses and a General Linear Model (ANCOVA) were estimated, controlling for age, educational level, and participation in social organisations; multiple linear regression models were additionally used as robustness checks. The results indicate that environmental collective action willingness constitute the strongest and most robust correlate of positive evaluations of the social outburst, followed by political identification, with respondents identifying with the left reporting more favourable perceptions than those identifying with the right. In contrast, attitudes toward democracy and gender do not exhibit independent effects in the fully adjusted model, while age introduces a consistent generational gradient. Overall, the findings suggest that perceptions of the Chilean social outburst are structured primarily by dispositional orientations toward collective action and, to a lesser extent, by ideological positioning, rather than by normative evaluations of democracy.

## Introduction

Over the past decade, a transnational cycle of mobilization has emerged, challenging political representation, social inequalities, and the quality of democracy. In diverse sociopolitical contexts, mass protests—from the Arab Spring and the European Indignados to Euromaidan, the “Yellow Vests,” Hong Kong, India, and Black Lives Matter—have combined discontent with inequality, perceptions of injustice, and institutional disaffection with demands for greater participation (Bringel and Pleyers, 2017; Klein Bosquet, 2012; Garduño García, 2014; Karyotis and Rüdig, 2017; Otálora Sechague, 2019; Purbrick, 2019; Mahmudabad, 2020; Gurcan and Donduran, 2021; Núñez Cruz, 2021). In Latin America, large-scale protests in Brazil (2013), Venezuela (2014, 2017, 2019), Ecuador (2019, 2022), Colombia (2019, 2021), and Bolivia (2019) were organized around issues of inequality, corruption, economic reforms, and a generalized crisis of representation (Fair, 2021; Pereira and Sabbagh Fajardo, 2023; Coronel and Donoso, 2024). These waves—often referred to as “social outbursts”—share an intensive use of digital networks to organize and build collective identities, as well as activism developed outside of political parties. However, they have shown ambivalent effects: in some cases enabling democratizing openings, and in others, authoritarian regressions through repression and the closing of civic spaces (Jost et al., 2018; Asún et al., 2020; García-González and Bailey, 2021; Masri, 2017; Friz, 2021; Korany and El-Mahdi, 2012; Conde, 2017; King, 2022).

Although embedded within a global wave of demands for social justice, recognition, and democratic deepening, the 2019 Chilean social outburst constitutes a paradigmatic case. Triggered by a rise in public transportation fares, it rapidly evolved into a structural challenge to the inequalities of the socioeconomic model and the legitimacy foundations of the post-dictatorial political system (Jiménez-Yañez, 2020; Friz, 2021). This complexity has stimulated the development of an expanding academic corpus addressing its origins, dynamics, and outcomes, through approaches that intersect sociology, political science, communication studies, culture, and gender (Fair, 2021; Coronel and Donoso, 2024; Joignant and Garrido-Vergara, 2025). It should also be noted that the Chilean social outburst is part of a broader cycle that, since 2011, has been marked by the expansion of protests from various social movements that, at both national and subnational scales, have questioned the social order, neoliberalism, and post-dictatorial politics.

Phenomena of this magnitude are of particular relevance, as mass protests—such as those in Chile, which were met with strong police repression—can reshape a country’s institutional trajectory, as occurred in Chile where the social outburst led to a constituent process aimed at replacing the constitution inherited from the dictatorship (Heiss, 2021), and reveal structural tensions around social justice, democracy, and human rights (Carrasco Paillamilla and Disi Pavlic, 2023). Understanding how citizens perceive these uprisings is essential, as such perceptions directly influence the legitimacy attributed to protest as a mechanism for social change and shape the political system’s subsequent responses, whether in the form of support for reforms or endorsement of repressive policies (Carrasco Paillamilla and Disi Pavlic, 2023). Furthermore, 5 years after the Chilean social outburst, ongoing disputes over its social memory—largely centered in the capital—highlight the importance of understanding perceptions of this event at subnational scales.

From a subnational perspective, the Valparaíso Region constitutes a particularly relevant case for analyzing perceptions of the Chilean social outburst, insofar as it concentrates historical and structural processes that have persistently shaped social and political conflict in the country. Its trajectory as a port city and early economic hub generated cycles of expansion and decline that produced a highly unequal socio-territorial structure, marked by deindustrialization, urban deterioration, and economic restructuring during the neoliberal period (Seoighe and Cuevas Valenzuela, 2021). This trajectory is further compounded by the close relationship between port-related economic activity, logistics, and socio-environmental impacts, which has reinforced patterns of uneven territorial development (Cuevas Valenzuela et al., 2023). In this context, the region exhibits persistent territorial inequalities and a strong culture of social mobilization, made visible by the symbolic centrality acquired by Valparaíso’s urban space during the 2019–2020 protest cycle, including disputes over symbols of political power such as the National Congress (Ferrada Aguilar, 2021). Taken together, these features make Valparaíso a particularly sensitive setting for examining how citizens interpret the social outburst.

Consistent with this perspective, an incipient but growing body of research has used public opinion data from Valparaíso precisely because of its analytical value as a subnational laboratory for studying social discontent, territorial inequalities, and democratic attitudes. Empirical evidence shows that the region displays territorially differentiated patterns of dissatisfaction and feelings of abandonment, linked not only to individual-level factors but, crucially, to conditions of the area of residence (Fierro et al., 2024). Complementarily, research has demonstrated that center–periphery divisions and territorial inequalities are associated with gaps in political efficacy and digital participation, underscoring the importance of studying the region to understand how territory structures political attitudes (Fierro et al., 2021).

In summary, analyzing citizens’ perceptions of contemporary uprisings provides an essential avenue for assessing democratic vitality and understanding the psychosocial mechanisms that shape the legitimacy of protest. Examining how different explanatory factors influence these perceptions—specifically, collective action disposition, attitudes toward democracy, political identification, and gender—allows for a deeper understanding of political behavior and the ways in which individuals interpret disruptive sociopolitical events. These dimensions are particularly relevant because they integrate evaluative, ideological, and identity components that influence how citizens construct meaning and legitimacy around processes of social mobilization.

### Social outbursts and empirical evidence on their perception

Mass protests—or social outbursts—have become a defining form of political conflict in the 21st century. Unlike armed confrontations, these uprisings consist of the collective action of unarmed citizens challenging established power through marches, strikes, occupations, and other forms of civil resistance (Chenoweth, 2020). In many recent cases, such protests have emerged within democratic contexts, articulating unmet demands from within the institutional system. For example, the massive Chilean mobilizations of 2019 were initially triggered by an increase in metro fares, which operated as a catalytic event rather than as the structural cause of the social outburst. A growing body of research agrees that this episode activated long-standing accumulated grievances linked to persistent inequalities and to widespread perceptions of abuse associated with the neoliberal model (Araujo and Martuccelli, 2012; Somma et al., 2021; Carrasco Paillamilla and Disi Pavlic, 2023). Similarly, countries such as Ecuador, Colombia, and Peru have experienced “outbursts” motivated by rising living costs and accumulated grievances, indicating a regional pattern of social uprisings as expressions of latent conflicts within Latin American democracies (Sehnbruch and Donoso, 2020; Doran, 2017).

At a comparative level, social outbursts tend to occur when traditional mechanisms of representation fail to channel citizens’ demands, resulting in sudden, spontaneous uprisings that initially garner broad popular support (Carrasco Paillamilla and Disi Pavlic, 2023). This contemporary form of conflict poses significant challenges: on the one hand, it can open opportunities for profound political transformations (e.g., democratic transitions following successful nonviolent protests; Bayer et al., 2016); on the other, it carries risks of disorder and state repressive responses that may erode public trust (Metcalfe and Pickett, 2022). In Chile, the 2019 social outburst led to unprecedented levels of human rights violations committed by security forces, which generated intense anger and indignation among the population toward such violence (Hatibovic et al., 2023), recalling the events that occurred during the military dictatorship (1973–1990). These dynamics illustrate how social outbursts have become a central form of sociopolitical conflict today, whose outcomes largely depend on the perceptions and attitudes they elicit among different sectors of society.

A growing body of international literature has examined citizens’ perceptions of protests and social uprisings, identifying several influential factors. First, individual predisposition toward collective action emerges as a key determinant. People with a history of activism tend to view protests more favorably and to legitimize their causes more frequently (Thomas and Louis, 2013; Sabucedo et al., 2018). Direct participation in mobilizations produces biographical effects that enhance subsequent political engagement (Carrasco Paillamilla and Disi Pavlic, 2023). For instance, studies in Chile found that those who participated in the 2019 social outburst developed a sharper awareness of injustice and were more likely to believe in protest efficacy as a means of change (Carrasco Paillamilla and Disi Pavlic, 2023; Castro-Abril et al., 2021). Even indirect exposure can be relevant: Bursztyn et al. (2021) demonstrated that social interaction with activist peers during protests has lasting effects on future political participation. Moreover, individuals who believe in climate change are more inclined toward collective action than those who do not (Hatibovic et al., 2025). Consistently, individuals with strong universalist moral values or a global identity—traits associated with participation in transnational causes such as environmentalism—tend to be more supportive of protests against perceived injustices (Carrasco Paillamilla and Disi Pavlic, 2023). This suggests that a pro-activism orientation (whether environmental or otherwise) predisposes individuals to interpret social uprisings as legitimate and necessary, in contrast to those less engaged in collective action, who may perceive them more negatively.

Second, political identification and ideology consistently emerge as some of the most powerful determinants in shaping perceptions of protests. Left–right divisions tend to color the interpretation of these events: individuals aligned with progressive positions usually emphasize the structural causes of discontent (inequality, injustice) and legitimize protests as valid expressions of social demands, whereas those positioned on the political right more frequently discredit mobilizations, viewing them as irrational, violent, or instigated by radical minority groups (Dammert and Sazo, 2020; Sehnbruch and Donoso, 2020). Previous research on attitudes toward the U.S. civil rights movement indicated that conservative participants identifying as white from the southern states largely rejected those protests, primarily due to racial prejudice and a strong attachment to the existing social order, whereas more liberal individuals tended to support them (Andrews et al., 2016). Similarly, during the 2020 Black Lives Matter anti-racist protests, partisan identification produced sharp divides in perception: Democrats tended to view them as legitimate demonstrations against racial injustice, while Republicans focused on rioting and supported a strong police response (Wasow, 2020; Edwards and Arnon, 2021).

In Chile, surveys conducted after the 2019 social outburst revealed this ideological polarization. Results from the December 2019 CEP Survey showed a marked ideological divide in evaluations of the October protests. While 66.7% of individuals identifying with the far left (1 on a 1–10 ideological scale) reported supporting the demonstrations, only 27.6% of those on the far right (value 10) expressed the same support. Conversely, 55.6% of right-leaning respondents rejected the protests, compared with only 8.8% of those on the left. These figures reflect a clear ideological division in the interpretation of the social outburst: left-wing sectors tended to evaluate it positively, whereas right-wing respondents generally adopted a critical view focused on public order and violent incidents [Centro de Estudios Públicos (CEP), 2019]. Likewise, academic research has shown that authoritarian or order-oriented beliefs (more prevalent among conservative groups) correlate with lower tolerance toward disruptive protest and greater support for repression (Krause, 2022; Gerber and Jackson, 2017). In contrast, individuals endorsing egalitarian and human rights values are more likely to defend the right to protest, even in contentious contexts (Crowson and DeBacker, 2008; Carrasco Paillamilla and Disi Pavlic, 2023). In sum, political-ideological identity functions as a powerful interpretive lens: partisan loyalties and identities shape whether an individual perceives a popular uprising as the legitimate “voice of the people” or as a threat to democratic order.

Third, attitudes toward democracy and institutions significantly shape perceptions of protests. Numerous studies indicate that individuals dissatisfied with the functioning of democracy or distrustful of institutions tend to view mass mobilizations as understandable—or even legitimate—means to push for change (Ketchley and El-Rayyes, 2021; Grande and Saldivia Gonzatti, 2025). In contexts where sectors of the public perceive a democratic deficit—such as corruption, exclusion, or lack of governmental responsiveness—they are more likely to interpret a social outburst as “the people making their voices heard” rather than mere disorder (Andrews et al., 2016). Comparative evidence reinforces this interpretation. In post-Mubarak Egypt, the prolonged cycle of protest between 2011 and 2013 weakened trust in liberal democratic institutions among some citizens, while simultaneously strengthening demands for greater political participation and renewal among others (Ketchley and El-Rayyes, 2021). Similarly, comparative evidence from Latin America indicates that support for democracy does not necessarily inhibit protest but can coexist with contentious orientations. Analyses based on Latinobarómetro data show that strong normative support for democracy, coupled with critical evaluations of its performance, is associated with a higher willingness to engage in protest (Cuevas and Villalobos, 2017). This pattern aligns with the notion of “critical democrats,” which helps explain how democratic support and social mobilization can coexist in contexts such as Chile.

In Chile, the social outburst occurred after years of declining institutional trust and growing dissatisfaction with political representation, creating conditions in which broad sectors perceived protest as a legitimate mechanism for correcting the national course (Somma et al., 2021; Argote and Visconti, 2025). Indeed, local studies show that disillusionment with political parties and self-identification as “independent/anti-elite” were positively correlated with justifying the 2019 mobilizations (Argote and Visconti, 2025). At the same time, there is evidence that strong normative democratic values also play a role: individuals with a high attachment to democratic principles may support the right to peaceful protest as an essential component of democracy (Carrasco Paillamilla and Disi Pavlic, 2023), even while rejecting violent acts. This complex relationship suggests that both democratic disaffection (which encourages seeing protest as necessary) and democratic commitment (which validates protest as civic expression) influence how a social outburst is evaluated, depending on the context and on whether institutions are perceived to have failed.

Fourth, several studies suggest that gender may play a role in how individuals perceive and participate in protests, although in more subtle and context-dependent ways. On one hand, gender differences exist in both participation and attitudes toward certain protest tactics: specifically, women tend to participate more frequently than men in non-confrontational protest activities, whereas men show a greater inclination toward forms of activism involving confrontational actions (Dodson, 2015). These differences may translate into distinct perceptions: some evidence indicates that women generally express lower support for violent tactics and a preference for nonviolent methods of protest (Gerber et al., 2023). On the other hand, recent experimental studies reveal that the gender composition of protests influences public reactions. Naunov (2025) found that protests with higher female participation tend to be perceived as less violent and less deserving of repression compared to male-dominated protests. However, this positive effect depends on gender stereotypes: when female protesters are portrayed as “challenging patriarchy” (e.g., radical feminists), the public tends to view them with greater moral suspicion and to justify repression against them more readily; conversely, when women protest while emphasizing traditional roles (as mothers or wives), they elicit greater sympathy and credibility (Naunov, 2025). These findings highlight that gender not only shapes participation in protests but also influences their social interpretation: public imaginaries of a movement’s “femininity” or “masculinity” can affect whether it is perceived as legitimate or threatening. In the Chilean case, the social outburst featured a strong female presence, including the iconic feminist performance “*A Rapist in Your Path*”, which may have partially mitigated perceptions of violence within the movement and linked it to gender-related claims. Nevertheless, the literature suggests continuing to explore gender-based differences in protest evaluations—for example, whether there are male–female gaps in justifying violence by either protesters or the police (Gerber et al., 2023)—since findings so far appear to depend on contextual factors and on the roles socially attributed to each gender in contentious settings. Recent literature shows that gender structures dispositions toward protest through processes of political socialization and differentiated normative frameworks. In Chile, the feminist cycle initiated in 2018 reconfigured repertoires of collective action, privileging more performative and less confrontational forms, which contributed to broadening the social legitimacy of protest and resignifying its public meaning (Disi Pavlic et al., 2024). Comparative studies indicate that these transformations are associated with differentiated patterns of participation in and evaluation of protest among women and men, both in Latin America and in other democratic contexts (Coffé and Bolzendahl, 2010; Viterna, 2013).

Although recent empirical research has advanced in identifying factors associated with perceptions of protests, significant gaps remain. Few studies have integrated variables such as predisposition toward collective action, attitudes toward democracy, political identification, and gender. Most have focused on isolated factors—such as participation in protests or ideological polarization (Carrasco Paillamilla and Disi Pavlic, 2023)—without providing a comprehensive explanatory model. At the international level, case studies make it difficult to draw general conclusions about the profiles of support or rejection toward social uprisings. This gap is particularly relevant in the Chilean post-2019 context, where initial support for the social outburst has declined over time (Activa Research, 2024), yet it remains unclear whether this variation reflects generational factors, media narratives surrounding violence (Edwards and Arnon, 2021), or contextual effects such as the pandemic. Therefore, an integrative approach is needed to understand how multiple factors interact to shape public perceptions of large-scale protests.

To address this gap, the present study examines four complementary dimensions that capture different levels of structuring of perceptions regarding the Chilean social uprising. First, a dispositional dimension refers to individuals’ willingness to engage in collective action, reflecting their openness to political mobilization. Second, an ideological dimension, represented by political identification, organizes interpretations of social conflict along the left–right spectrum. Third, a normative dimension captures attitudes toward democracy as a political regime. Finally, a social-position dimension, represented by gender, reflects cleavages that have been particularly salient in recent cycles of mobilization.

Taken together, these dimensions allow the articulation of dispositions toward mobilization, ideological orientations, normative evaluations of the political order, and social cleavages that structure contemporary political experience. While other potentially relevant determinants exist—such as institutional trust, partisanship, socioeconomic resources, or media exposure—these fall outside the scope of the present analysis. The study instead adopts a parsimonious analytical strategy centered on dispositional, ideological, normative, and identity-related dimensions that the literature has identified as especially influential in shaping attitudes toward protest.

### Objectives and hypotheses of the study

This study seeks to address existing gaps through a systematic analysis of citizens’ perceptions of the 2019 Chilean social outburst as a dependent variable, integrating factors such as collective action disposition, democratic attitudes, political identification, and gender. This approach allows for a deeper understanding of the psychosocial and political mechanisms that lead different groups to legitimize or reject a mass protest. Theoretically, the study contributes to social psychology by examining how citizens’ prior characteristics influence their evaluation of conflict, complementing research that has explored the effect of protest on political consciousness (Vestergren et al., 2017; Pop-Eleches et al., 2022). Practically, understanding the profiles of support or rejection has relevant implications for democratic governance and the design of public policies in polarized contexts. Thus, the research is justified both by its academic contribution and by its practical value in strengthening institutional responses to future social crises.

Within this framework, the objective of this study is to determine how the disposition to participate in collective actions (including environmental activism), attitudes toward democracy, political identity (self-placement on the ideological spectrum), and the respondent’s gender explain their evaluation of the social outburst.

Using bivariate and multivariate analytical models, the following hypotheses derived from the literature were tested. It is important to clarify that H4 includes a partially exploratory component, given that empirical evidence on the relationship between gender and evaluations of the social outburst remains limited:

*H1.* A greater disposition to engage in environmental collective action will be associated with more favorable evaluations of the Chilean social outburst, even after controlling for political identification, attitudes toward democracy, and relevant control variables.

*H2.* Political identification will be associated with systematic differences in perceptions of the social outburst. Specifically, individuals who identify with the political left are expected to evaluate the outburst more positively than those who identify with the right, while individuals without political identification are expected to display differentiated evaluations, once the remaining variables in the model are controlled for.

*H3.* Attitudes toward democracy will be associated with differences in perceptions of the Chilean social outburst, even after controlling for dispositions toward environmental collective action, political identification, and control variables. Specifically, individuals who consider democracy to be preferable to any other form of government are expected to evaluate the social outburst more positively than those who justify an authoritarian government under certain circumstances or express indifference toward the type of regime.

*H4.* Gender will be associated with differences in perceptions of the Chilean social outburst, even after controlling for dispositions toward environmental collective action, political identification, attitudes toward democracy, and sociodemographic variables. Specifically, women are expected to evaluate the social outburst more positively than men, once the remaining variables included in the model are taken into account.

In summary, this study aims to provide empirical evidence on the psychosocial and political determinants that shape citizens’ evaluations of the Chilean social outburst. To test the proposed hypotheses and examine the relationships among the selected variables, a methodological design was developed to assess, in an integrated manner, the relative weight of each factor in shaping perceptions of the conflict.

## Methodology

This study employed a correlational survey design, non-experimental and cross-sectional in nature (Hernández et al., 2010). Fieldwork and data collection were conducted between July 22 and September 21, 2024, under the supervision of the consulting firm H&N Servicios Integrales Spa.

### Sampling

The survey was administered using a probabilistic and stratified sampling design with a 95% confidence level and a margin of error below ±4%, assuming maximum variance (*P*−*Q* = 0.5). The final sample consisted of 809 residents aged 18 years or older from the continental area of the Valparaíso Region. Stratification was based on population distribution by: (1) Provincial capitals—Los Andes, La Ligua, Quillota, San Antonio, San Felipe, Valparaíso, and Quilpué; and (2) Communes with populations exceeding 100,000 inhabitants—Viña del Mar and Villa Alemana.

Subsequently, each commune was divided into geographic zones (north, south, east, and west) using the main square as a reference point, except for San Antonio, which was organized into two zones due to its territorial configuration. In communes such as Valparaíso, an additional criterion of socioeconomic vulnerability was incorporated to ensure balanced territorial coverage.

Finally, within each zone, neighborhoods, blocks, households, and individuals were selected through random procedures, ensuring representativeness by territory, sex, and age. This multistage procedure allowed for a balanced sample proportional to the regional population distribution.

Data collection was conducted through face-to-face interviews using paper-based questionnaires administered by trained interviewers from the consulting firm H&N Servicios Integrales Spa. Fieldwork was carried out between July 22 and September 21, 2024, by an average team of 13 interviewers, following a probabilistic, stratified, and multistage sampling design. Surveys were considered valid only if all items were completed, informed consent was obtained, eligibility criteria were met, and fieldwork verification records were available. Data were digitized in parallel, and quality control procedures included the exclusion and replacement of six invalid questionnaires.

### Participants

The study included 809 participants with a mean age of 47.00 years (SD = 17.1), of whom 52.7% were women, 46.7% were men, and 0.5% identified with another gender. Participants reported having lived in the region for an average of 34.8 years (SD = 20.0).

Most respondents were married (37.6%), followed by single individuals (33.0%), while the remainder were distributed among cohabiting partners, divorced, widowed, and a small percentage who did not provide a response. In terms of education, the majority reported having completed secondary education (33.5%) or higher education—technical or university (34.9% combined)—while smaller proportions had only completed primary education, pursued postgraduate studies, or had no formal education. Regarding occupation, most participants were salaried employees (40.4%), followed by self-employed workers (19.2%) and retirees or pensioners (19.2%), while smaller groups were engaged in unpaid domestic work, studying, or unemployed. Overall, the results describe a population with medium to high educational attainment and broad participation in the labor market.

### Ethical considerations

Participants provided informed consent prior to participation. The consent form stated that participation was entirely voluntary, unpaid, and confidential. No personal data allowing for participant identification were collected, and the results were used exclusively for academic purposes. The databases were securely stored and safeguarded by the principal investigator of the study.

### Measures

*Perceptions of the social outburst*. This variable was measured using a four-item scale designed to assess the level of agreement with various statements regarding the effects and meanings of the Chilean social outburst. The items were: (1) “The social outburst made visible various problems affecting Chilean society,” (2) “The social outburst was necessary for Chile’s development,” (3) “The social outburst promoted or contributed to significant changes in Chile,” and (4) “The social outburst facilitated the expression of different social groups (students, sexual diversities, workers, retirees, etc.).” Responses were recorded on a 7-point Likert-type scale (1 = strongly disagree, 7 = strongly agree), where higher scores indicated a more positive perception of the phenomenon. The scale showed good internal consistency (*α* = 0.85).

*Willingness to engage in environmental collective action* (adapted from Sandoval et al., 2018). An adapted version of a collective action scale was used, incorporating environmental concerns into each item. Participants were asked to indicate, on a scale from 1 (not at all willing) to 7 (extremely willing), the extent to which they would be willing to engage in various actions. The items were: “Participate in legal/authorized demonstrations (marches) in favor of environmental protection and/or against pollution,” “Support boycotts against polluting companies,” “Take part in strikes for environmental protection,” “Occupy buildings or factories (sit-ins) for environmental protection,” “Participate in barricades or destruction for environmental protection,” “Vote in municipal, parliamentary, or presidential elections for candidates supporting environmental protection,” “Sign a petition in favor of the environment,” and “Share my opinions about politics on social media (Twitter, Facebook, etc.) in favor of environmental protection.” The scale demonstrated good internal consistency (*α* = 0.83).

*Political identification*. Political identification was measured using a self-placement ideological scale ranging from 1 (left) to 10 (right). In light of the sustained process of depoliticization and the weakening of the left–right axis in the Chilean context, as well as low levels of party identification and the limited institutional presence of a clearly defined political center, the intermediate scale values (5 and 6) were recoded as “no political identification.” This decision was intended to avoid the analytical overrepresentation of an ideological center lacking a clear empirical counterpart in the current political system, and to treat these positions instead as reflecting a relative equidistance from the left and the right rather than a substantive identification with a defined political center.

*Attitude toward democracy*. This was measured using a single-choice question: “Which of the following statements about democracy do you agree with the most?” The response options were: (1) “Democracy is preferable to any other form of government,” (2) “Under some circumstances, an authoritarian government can be preferable to a democratic one,” and (3) “For people like me, it makes no difference whether the government is democratic or authoritarian.”

*Gender.* Gender identity was assessed using a single-choice question: “Please indicate your gender identity,” with three response options: female (1), male (2), and other (3). For statistical analysis, given that the “other” category included a very small number of respondents (*n* = 4; 0.5%)—insufficient to form a comparative group for mean analyses—these cases were treated as missing values, and the variable was retained in dichotomous form (female/male).

*Control variables*. Age, educational attainment, and social organizational participation were included as control variables. Age was treated as a continuous variable. Educational attainment was measured as the highest level of education completed and recoded into ordered categories reflecting increasing formal education, excluding “do not know” and “no response” categories. Social organizational participation was assessed by asking respondents about their active involvement in different types of organizations and recoded into a dichotomous variable distinguishing between those who participate in at least one organization (1) and those who do not participate in any (2).

### Data analysis

**Figure 1:**
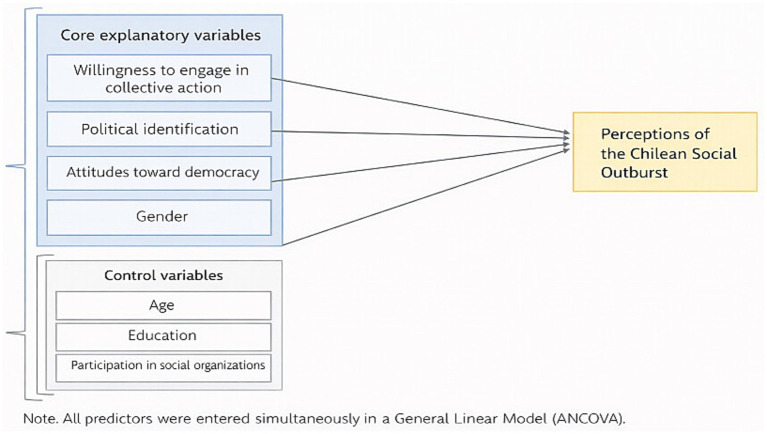
General Linear Model (ANCOVA) predicting perceptions of the Chilean social outburst. The figure illustrates the analytical structure of the General Linear Model (ANCOVA). Core explanatory variables and control variables were entered simultaneously to estimate their independent association with perceptions of the Chilean social outburst.

Descriptive statistics were used to summarize the data. A General Linear Model (GLM/ANCOVA) was estimated to examine differences in perceptions of the social outburst, including dispositions toward environmental collective action, political identification, attitudes toward democracy, and gender as explanatory variables, and age, education, and participation in social organizations as controls (see Figure 1). Multiple linear regression models were additionally estimated as robustness checks. All analyses were conducted using PSPP and Jamovi (version 2.3.21.0).

Given the probabilistic and stratified sampling design, no *a priori* power analysis was conducted. However, the final sample size provides adequate statistical power for multivariate analyses. With a significance level of *α* = 0.05, this sample size allows the detection of small to moderate effects in General Linear Models with multiple predictors. Moreover, the main effects reported in the study exhibit consistent and stable effect sizes, reducing the likelihood of Type II errors and supporting the robustness of the findings.

## Results

This section presents the main findings of the study. First, descriptive statistics summarize participants’ characteristics and the distribution of key variables. Second, a General Linear Model (ANCOVA) is estimated to examine differences in perceptions of the social outburst while controlling for age, education, and participation in social organizations. Finally, multiple linear regression models are reported as robustness checks to assess the stability of the results across analytical strategies.

### Descriptive analyses

First, a descriptive analysis was conducted for the variables included in the study. Perception of the social outburst showed a mean of *M* = 4.72 (*SD* = 1.69), with values ranging from 1 to 7, indicating a generally moderate and favorable tendency toward the phenomenon. Regarding willingness to engage in environmental collective action, the variable presented a mean of *M* = 4.16 (*SD* = 1.59). This result suggests a moderate level of willingness to engage in environmental collective actions, reflecting an intermediate trend in citizens’ environmental commitment.

In relation to attitudes toward democracy, the majority of participants considered democracy preferable to any other form of government (54.8%), followed by those who were indifferent (29.1%), and, to a lesser extent, those who justified an authoritarian government under certain circumstances (16.1%).

As for political identification, 24.0% of participants identified with the left, 58.2% reported no political identification, and 17.8% positioned themselves on the right. Finally, the gender variable showed a relatively balanced distribution, with 53.0% women and 47.0% men.

These results indicate a sample predominantly composed of participants expressing moderate levels of collective action disposition, democratic preference, no defined political identification, and a slight female majority.

### General Linear Model (ANCOVA)

A General Linear Model (ANCOVA) was estimated to analyze perceptions of the Chilean social outburst, simultaneously including dispositions toward collective action, political identification, attitudes toward democracy, and gender as core explanatory variables, while age, educational level, and political participation were incorporated as control variables (see Table 1).

**Table 1:** Results of the General Linear Model (ANCOVA) for perceptions of the Chilean social outburst.

Predictor	df	*F*	*p*	*η* ^2^ *p*	*ω* ^2^
Collective action (environmental)	1	52.09	<0.001	0.090	0.082
Political orientation	2	11.02	<0.001	0.040	0.032
Support for democracy	2	0.23	0.795	0.001	−0.002
Gender	1	0.75	0.388	0.001	0.000
Age	1	10.72	0.001	0.020	0.016
Education (recoded)	1	2.20	0.139	0.004	0.002
Political participation (recoded)	1	0.97	0.325	0.002	0.000
Political orientation × Gender	2	1.33	0.266	0.005	0.001
Democracy × Gender	2	0.14	0.866	0.001	−0.003
Democracy × Political orientation	4	0.22	0.929	0.002	−0.005
Democracy × Gender × Political orientation	4	0.40	0.809	0.003	−0.004
Model	21	6.21	<0.001	—	—

The overall model was statistically significant, *F*(21, 526) = 6.21, *p* < 0.001, indicating that the set of included variables explains a significant proportion of the variance in perceptions of the social outburst. The inclusion of control variables further strengthens the robustness of the model.

### Main effects of the core explanatory variables

Disposition toward participation in environmental collective action emerged as the strongest predictor in the model, *F*(1, 526) = 52.09, *p* < 0.001, with a moderate effect size (*η*^2^*p* = 0.090; *ω*^2^ = 0.082). This effect remained significant after controlling for age, educational level, and political participation, as well as for political identification, attitudes toward democracy, and gender. Taken together, this result indicates that perceptions of the social outburst are primarily structured by general dispositions toward collective action rather than by normative attitudes or sociodemographic attributes.

Political identification also showed a significant and independent effect on perceptions of the social outburst, *F*(2, 526) = 11.02, *p* < 0.001, with a small-to-moderate effect size (*η*^2^*p* = 0.040; *ω*^2^ = 0.032). This effect persisted after controlling for age, educational level, and political participation, confirming that the social outburst retains a relevant ideological dimension, although one that is subordinate to the explanatory weight of collective action dispositions.

### Explanatory variables without independent effects

Attitudes toward democracy did not show a significant effect in the fully adjusted model, *F*(2, 526) = 0.23, *p* = 0.795, with a virtually null effect size (*η*^2^*p* ≈ 0.001). This result suggests a decoupling between normative support for democracy and evaluations of the social outburst once activist dispositions and sociodemographic controls are taken into account.

Similarly, gender did not present a significant effect, *F*(1, 526) = 0.75, *p* = 0.388, *η*^2^*p* ≈ 0.001. The absence of a direct gender effect, even after controlling for age, educational level, and political participation, indicates that gender likely operates indirectly through more proximal dispositional and ideological variables.

### Control variables

Among the control variables, age showed a significant effect, *F*(1, 526) = 10.72, *p* = 0.001, with a small effect size (*η*^2^*p* = 0.020), indicating the presence of generational differences in evaluations of the social outburst. In contrast, recoded educational level did not reach statistical significance, *F*(1, 526) = 2.20, *p* = 0.139, *η*^2^*p* = 0.004, nor did political participation, *F*(1, 526) = 0.97, *p* = 0.325, *η*^2^*p* = 0.002. Importantly, the inclusion of these control variables did not reduce the substantive effect of dispositions toward environmental collective action, ruling out the possibility that this result merely reflects higher levels of general political involvement or basic structural differences.

No significant effects were observed for any of the tested interactions (democracy × gender, democracy × political orientation, gender × political orientation, or the three-way interaction), indicating that the effects of the core explanatory variables operate in an additive and independent manner.

Regarding model assumptions, Levene’s test indicated a mild violation of the homogeneity of variance assumption, *F*(17, 530) = 1.71, *p* = 0.038. However, given the sample size (*N* ≈ 550), the General Linear Model is robust to this type of deviation. The Shapiro–Wilk test of normality was significant, *W* = 0.990, *p* < 0.001, as expected in large samples; visual inspection of the Q–Q plot indicated only minor deviations.

Overall, the results show that perceptions of the Chilean social outburst are primarily structured by dispositions toward collective action and, to a lesser extent, by political orientation, even after controlling for age, educational level, and political participation. Gender and attitudes toward democracy do not exert direct effects in the adjusted model, while age introduces a consistent generational gradient. The model proves robust to changes in specification, reinforcing the strength of the empirical argument.

### Multiple linear regression

To assess the robustness of the main findings, we estimated a multiple linear regression model including the same set of predictors and controls used in the GLM. The results corroborate the central conclusions of the ANCOVA: dispositions toward environmental collective action emerged as the strongest predictor of perceptions of the social outburst, followed by political orientation and age, while support for democracy, education, and political participation showed no significant independent effects. Gender displayed a small but statistically significant coefficient in the regression model; however, this effect did not remain significant in the fully adjusted GLM, suggesting that gender differences are largely accounted for by dispositional and ideological variables rather than constituting an independent explanatory dimension. Overall, the convergence of results across analytical strategies indicates that the main conclusions are not sensitive to model specification.

## Discussion

This study provides empirical evidence that perceptions of the Chilean social outburst are primarily structured by dispositions toward collective action and, to a lesser extent, by political orientation, even after controlling for age, educational level, and participation in social organizations. In the adjusted model, neither gender nor attitudes toward democracy exert independent direct effects, while age introduces a consistent generational gradient. The stability of these findings across alternative model specifications reinforces the robustness of the empirical argument. Taken together, the absence of significant effects of attitudes toward democracy suggests that evaluations of the social outburst are shaped more by relatively stable dispositional and identity-based orientations—such as willingness to engage in environmental collective action and political identification—than by abstract normative evaluations of the democratic regime. Regarding gender, its lack of significance in the fully specified model indicates that observed differences operate indirectly, mediated by processes of political socialization and orientations toward collective action, rather than functioning as an autonomous explanatory cleavage in evaluations of the social outburst.

Regarding the testing of the study’s hypotheses, the results partially confirmed the proposed expectations and allow articulation with the theoretical evidence reviewed on citizens’ perceptions of social protests.

First, *H1*, which posited a positive association between willingness to engage in environmental collective action and a favorable perception of the social outburst, was clearly supported. The significant correlation and positive regression effect suggest that individuals more inclined to participate in collective causes—even in seemingly distinct domains such as environmental issues—tend to more strongly legitimize social mobilizations. This result is consistent with literature linking pro-activist orientations and universalist values with greater empathy toward protest movements (Thomas and Louis, 2013; Sabucedo et al., 2018; Carrasco Paillamilla and Disi Pavlic, 2023). In this sense, the finding reinforces the notion that dispositions toward collective action shape a transversal moral and motivational framework from which people interpret social uprisings as legitimate expressions of demands for social justice.

Second, *H2*, referring to the differentiating role of political identification, was also confirmed. The analyses showed a clear ideological gradient in the evaluation of the outburst: participants identifying with the left expressed significantly more positive perceptions than those without political affiliation and, to a lesser extent, than those identifying with the right. This pattern aligns with robust empirical evidence situating ideology as a central axis in the legitimation or delegitimation of protest (Wasow, 2020; Gerber and Jackson, 2017). As documented in Chile and other contexts, individuals with progressive positions tend to interpret mobilizations as legitimate responses to structural injustice, whereas those on the right emphasize their disruptive potential and associate them with disorder (Sehnbruch and Donoso, 2020; Krause, 2022). The magnitude of this effect in the multivariate model confirms that political identity operates as an ideological lens structuring interpretations of social conflict.

Regarding *H3* and *H4*, the results do not provide support for these hypotheses in the fully adjusted multivariate model. Attitudes toward democracy did not exhibit a significant independent effect on perceptions of the social outburst once ideological positioning and dispositional orientations toward collective action were taken into account. This finding is consistent with comparative research showing that democratic support can coexist with diverse and even opposing evaluations of protest, particularly in contexts marked by broad normative consensus around democracy (Andrews et al., 2016; Ketchley and El-Rayyes, 2021).

Similarly, *H4* was not supported in the fully specified ANCOVA model. Although gender displayed a small association with perceptions of the social outburst in robustness checks based on multiple linear regression, this effect did not remain significant once political identification, collective action dispositions, and sociodemographic controls were included in the General Linear Model. This pattern suggests that gender does not operate as an autonomous predictor of perceptions of the social outburst, but rather exerts an indirect influence through ideological orientations and dispositions toward collective action.

### Willingness to engage in environmental collective action: an activist transfer effect

The results of this study confirm psychosocial patterns consistent with international literature on collective action and attitudes toward protest. In particular, willingness to engage in environmental collective action was associated with a favorable evaluation of the social outburst. This finding introduces a novel perspective by suggesting an activist transfer or generalization effect across different domains of mobilization. Individuals predisposed to participate in pro-environmental causes also tended to view sociopolitical protest as more legitimate, reflecting the existence of a cross-cutting activist identity.

The literature has shown that identification with environmental movements extends beyond specific ecological behaviors, fostering a broader orientation toward social change and collective justice (Fielding et al., 2008). Environmental collective action often highlights the links between ecological crises, social inequalities, and the political and economic structures that sustain them, demanding that state responses address the multiple layers of harm to ecosocial justice (Luque-Lora, 2021). In this vein, our results suggest that values of solidarity, equity, and collective efficacy associated with environmental activism may extend to the legitimation of other forms of collective action. Historically, the Chilean context supports this interpretation. During the 2010s, student, environmental, and feminist movements consolidated a generation of actors with organizational and protest experience (Plaza and Rozas, 2024). This web of interconnected activist networks likely provided the cultural foundation that facilitated sympathy toward the social outburst. Therefore, willingness to engage in environmental collective action reflects not only a thematic commitment but also a broader civic orientation and critical stance toward the existing social order.

Moreover, when focusing on the Valparaíso Region, dispositions toward environmental collective action can be understood in light of the growing centrality of socio-environmental conflicts in the articulation of social discontent. In Chile, territorial disputes associated with extractivism—particularly around water—have ceased to be isolated local conflicts and have become politicized processes that challenge both the development model and territorial inequalities (Delamaza et al., 2017; Rivera et al., 2016). These conflicts express not only environmental damage but also social and territorial injustices, which has facilitated the incorporation of environmental demands into broader critiques of the neoliberal order (Campos-Medina, 2015). In this context, environmental collective action operates as a key interpretive framework for understanding the social outburst and its territorial expressions (Berasaluce et al., 2021).

The explanatory weight of willingness to engage in environmental collective action can be interpreted in light of the cross-cutting character that socio-environmental conflicts have acquired in Chile over the past decade. Several studies show that conflicts associated with extractive, energy, and territorial projects have generated intense forms of social mobilization and collective protest across different regions of the country (Ciftci and Lemaire, 2023; Dame et al., 2023). In this context, environmental activism is not limited to a specific thematic agenda; rather, it has become articulated with broader territorial, social, and political demands, turning into a space for civic socialization and collective mobilization (Panez Pinto et al., 2023; Gillot and Rérat, 2024).

Likewise, the literature on social movements in Chile shows that participation in environmental protests often intersects with other cycles of social mobilization and with more general forms of political participation, including student protests and citizen movements (Scherman et al., 2015; Scherman et al., 2021). In this sense, willingness to participate in actions related to environmental issues can be understood as an indicator of a broader orientation toward participation in public conflicts and collective protest.

Furthermore, the measure used in the survey captures a broad repertoire of collective action—rather than a narrow indicator of environmental behavior or lifestyle—which reinforces its value as an empirical approximation of a broader disposition toward civic mobilization in this domain.

This finding expands the traditional explanatory framework focused on structural variables (such as income or socioeconomic position) by introducing a less explored attitudinal component: activist predisposition as the psychological basis for empathy with protest. This connection suggests that prosocial and environmental orientations may function as indicators of a committed civic identity predisposed to legitimize forms of collective action even outside their specific domain.

### Political identification and ideological polarization

Political identification emerged as a significant and independent factor in the General Linear Model, even after controlling for collective action dispositions and sociodemographic variables. This finding indicates that ideological orientations structure how citizens interpret the social outburst, consistent with comparative evidence showing that political identities shape interpretative frameworks in contexts of social and political conflict (Barker et al., 2021; Freitas-Monteiro and Prömel, 2024). In our case, individuals identifying with left-wing positions tended to view the outburst as a legitimate expression of social discontent, whereas those on the right evaluated it more negatively or critically.

These findings are consistent with evidence showing an ideological asymmetry in the legitimation of protest: left-leaning individuals typically perceive mobilizations as valid tools for social change, while conservatives tend to associate them with disorder or threats to the status quo (Jost et al., 2012; Jost et al., 2017). This ideological divide—widely observed in consolidated democracies—was reproduced in the Chilean case and has deepened in the years following the event. Recent surveys indicate that, by 2024, retrospective support for the outburst reached 55% among those identifying with the left and only 23% among right-wing adherents [Centro de Estudios Públicos (CEP), 2024].

From a theoretical standpoint, this division can be understood as the result of processes of political identification and system justification. Progressive orientations are associated with collectivized identities and greater sensitivity to social injustice (Simon and Klandermans, 2001; Becker and Tausch, 2015), whereas conservative orientations tend to prioritize stability and institutional order (Jost et al., 2012). Thus, perceptions of the social outburst are not solely explained by a rational evaluation of events but also by ideological frameworks that guide the moral and political interpretation of protest.

The Chilean case offers an illustrative example of this dynamic. Although the outburst began as a cross-cutting reaction to inequality, it gradually became a focal point of ideological polarization (Plaza and Rozas, 2024). This evolution aligns with research showing that large-scale social conflicts tend to reactivate pre-existing divisions regarding the legitimacy of collective action (Carrasco Paillamilla and Disi Pavlic, 2023). Hence, political identity operated as an interpretive filter shaping both the moral judgment and the perceived efficacy and legitimacy of the movement.

### Gender and perceptions of the social outburst

The multivariate analyses indicate that gender does not constitute a direct and independent predictor of perceptions of the Chilean social outburst once dispositions toward collective action, political orientation, and sociodemographic controls are included. While less adjusted analyses—such as multiple linear regression—suggest small gender differences, these effects do not persist in the fully specified General Linear Model, indicating that observed gender gaps are largely mediated by dispositional and ideological factors rather than by gender as an autonomous explanatory cleavage.

This does not imply that gender is irrelevant. Rather, gender appears to operate as an indirect and contextual structuring dimension. In Chile, the cycle of mobilization initiated in 2018, particularly the feminist wave, reshaped the cultural and political frames through which protest is interpreted. Feminist and gender-based mobilizations have privileged more contained and performative repertoires, contributing to the social resignification and increased legitimacy of protest (Disi Pavlic et al., 2024). In this context, women’s more favorable evaluations of the social outburst can be understood as the outcome of broader transformations in political socialization and activist dispositions, consistent with research showing that gender differences in protest attitudes tend to be expressed through moral, communitarian, and care-oriented frames rather than direct effects (Viterna, 2013; Moraga et al., 2021; Osorio-Parraguez et al., 2022).

### Attitudes toward democracy: consensus and critique

Unlike the previous variables, attitudes toward democracy did not show a significant effect on perceptions of the social outburst. This seemingly counterintuitive result can be understood in light of two complementary factors: the existence of a strong normative consensus around democracy and the emergence of what the literature has described as *critical democrats* (Norris, 2011). Comparative evidence from Latin America shows that support for democracy does not operate as an automatic inhibitor of protest; on the contrary, high normative endorsement of democracy can coexist with, and even foster, contentious orientations toward political action (Cuevas and Villalobos, 2017).

Surveys conducted in Chile consistently indicate that a large majority of the population prefers democracy over any other form of government (Latinobarómetro, 2024). This widespread consensus likely produces a ceiling effect, reducing the variance of democratic attitudes and limiting their explanatory power in multivariate models. At the same time, empirical research shows that citizens may strongly value democracy while simultaneously holding critical evaluations of its actual functioning, a combination that is associated with higher willingness to protest (Cuevas and Villalobos, 2017). In this sense, both supporters and critics of the social outburst mobilized democratic frames: the former interpreted it as a legitimate expression of popular sovereignty, while the latter portrayed it as a threat to institutional order.

This pattern is consistent with broader findings in the literature on political participation in established democracies, which demonstrate that democratic commitment does not preclude protest participation (Dalton et al., 2010). On the contrary, citizens with stronger democratic orientations may engage in collective action to demand greater representativeness, accountability, or social justice (Inglehart and Welzel, 2005; Norris, 2011). In the Chilean case, the constitutional process that followed the outburst can be interpreted as an attempt to institutionally channel this form of democratic discontent (Heiss, 2021). Accordingly, the absence of a direct relationship between democratic attitudes and perceptions of the social outburst should not be read as political indifference, but rather as evidence of a broad normative consensus over democracy accompanied by divergent understandings of how democracy ought to be enacted—one more participatory and transformative, and another more institutional and order-oriented.

### Limitations, strengths, and future research directions

While the findings of this study provide an integrative overview, certain methodological limitations must be acknowledged. First, the cross-sectional and correlational design prevents establishing causal relationships. Although it is suggested that activist predisposition influences perceptions of the social outburst, it is also plausible that a positive evaluation of the movement may, in turn, increase future willingness to mobilize. Longitudinal or quasi-experimental studies could help clarify the direction of these associations. On the other hand, a major strength of this research lies in the use of a probabilistic stratified sampling design, which ensures a representative selection of the target population. This enhances the external validity of the findings and allows the results to be generalized beyond the analyzed sample. Consequently, the observed associations can be interpreted with stronger and more reliable empirical grounding.

Second, the analyzed variables are based on self-reported measures, which may introduce social desirability bias. In a politically sensitive context, some participants may have moderated negative opinions toward the outburst or overestimated their adherence to democracy. Future studies could complement surveys with qualitative methods or implicit measures to capture more spontaneous attitudes. However, recent evidence challenges the presumed superiority of implicit measures over self-reports, concluding that the latter often provide more consistent and valid estimates in most research contexts (Corneille and Gawronski, 2024).

A third limitation concerns the temporal framework of measurement. Perceptions of the social outburst have evolved substantially since 2019. National surveys show that support for the movement declined from 55% in 2019 to 23% in 2024 (Pareja, 2024). Therefore, our data reflect a specific moment within this broader process of collective reinterpretation. Caution is advised when generalizing the results to other periods or contexts. However, this temporal delimitation also represents a strength, as it captures an analytically precise “snapshot” of a key stage in the evolution of social unrest and its public meanings. This provides a valuable empirical basis for future longitudinal comparisons or for tracking changes in the perceived legitimacy of protest over time.

Another limitation of this study is its cross-sectional design, which precludes establishing the directionality of the observed associations and leaves open the possibility of inverse or reciprocal relationships between political dispositions and perceptions of the social outburst. These dynamics should be examined in future longitudinal research. Additionally, some key variables—particularly political identification and attitudes toward democracy—were measured using single-item indicators, which may limit the sensitivity of the estimates; therefore, these findings should be interpreted with caution, and future studies should employ multidimensional scales.

Finally, future research could examine how other value orientations—such as feminism, humanitarianism, or community engagement—shape the legitimization of protest. Including these variables would broaden our understanding of the relationship between identities and support for collective action.

### General conclusions

Overall, the results indicate that evaluations of the 2019 Chilean social outburst were not random but structured by the interaction of activist dispositions, political identification, and gender-related orientations. Citizens interpreted the conflict through preexisting ideological and value-based frameworks rather than through abstract support for democracy, which remained a broadly shared normative consensus. At the same time, while the mechanisms of discontent shaping these evaluations operate at a national level, territorial context introduces substantive modulations in the meanings attributed to protest. The case of Valparaíso shows that perceptions of the social outburst are not homogeneous across space and that local trajectories of inequality and conflict generate differentiated interpretive frameworks (Aguilera and Espinoza, 2022). From a theoretical perspective, these findings underscore the importance of incorporating territorial dimensions into explanatory models of attitudes toward protest and collective action.

Moreover, the temporal distance between the 2019 social outburst and the data collection conducted in 2024 requires considering processes of political and ideological re-signification of the event. Recent evidence shows that, over time, the outburst has been reinterpreted in more ambivalent and polarized ways, combining readings that frame it as a legitimate expression of social discontent with critical narratives emphasizing violence, disorder, and its institutional consequences [Somma et al., 2021; Centro de Estudios Públicos (CEP), 2024]. Studies conducted after the constitutional cycle indicate that these retrospective evaluations are strongly mediated by narrative disputes, conflicting political memories, and divergent assessments of the outburst’s effects on Chilean democracy (Aguilera and Espinoza, 2022; Nuñez and Palomé, 2022). In this context, perceptions measured in 2024 do not reflect immediate reactions to the event but rather interpretations shaped by its institutionalization, subsequent political controversies, and ongoing public debate over its legacy, reinforcing the need to analyze the social outburst as a dynamic process of re-signification rather than as a static episode.

This study thus contributes to understanding how individual orientations shape citizens’ interpretations of events involving high levels of political conflict. Specifically, it demonstrates that political identity remains the most powerful axis of differentiation, while the willingness to engage in environmental collective action introduces a novel component linking thematic activism to the legitimization of social protest. Furthermore, the finding that women evaluated the outburst more positively than men suggests a meaningful cultural shift associated with the repositioning of the female gender in Chile’s public sphere.

Finally, these results carry implications for democratic strengthening. Understanding how ideological and activist convictions shape evaluations of protest can inform communication and dialogue strategies aimed at reducing polarization. In contexts of crisis, recognizing the legitimacy of different forms of collective expression—without criminalizing or idealizing them—represents an essential step toward consolidating a participatory and tolerant political culture.

## Data Availability

The raw data supporting the conclusions of this article will be made available by the authors, without undue reservation.
